# Reply to Comment on: Lung Perfusion Imaging with Technetium-99m-macroaggregated Albumin should be Combined with Contrast-enhanced Echocardiography for the Diagnosis of Hepatopulmonary Syndrome

**DOI:** 10.4274/mirt.galenos.2021.58561

**Published:** 2021-02-09

**Authors:** Majid Assadi

**Affiliations:** 1 Bushehr University of Medical Sciences, Bushehr Medical University Hospital, The Persian Gulf Nuclear Medicine Research Center, Department of Molecular Imaging and Radionuclide Therapy (MIRT), Bushehr, Iran

**Keywords:** Hepatopulmonary syndrome, technetium-99m-macroaggregated albumin, lung perfusion scintigraphy, right-to-left shunt, contrast-enhanced echocardiography

## Dear Editor,

We appreciate the authors for their interest and knowledgeable comments on our study (1). We completely agree with them on dividing the geometric mean of brain counts by 0.13 since the brain is presumed to receive 13% of the cardiac output (2). We have used this score for shunt calculation.

The relationship between brain uptake and quantitation of the right-to-left (R­-L) shunt percentage using technetium-99m (Tc-99m)-macroaggregated albumin (MAA) whole-body imaging has been rarely investigated. Ito et al. (3) studied 53 patients and found that Tc-99-MAA brain uptake could completely distinguish patients with or without an R-L shunt and that it could provide complementary information and appears promising in predicting clinical outcomes.

With our extensive experience in assessing R-L shunting as a routine adjunct protocol in a large number of patients presenting for ventilation/perfusion single photon emission computed tomography scans, in addition to those who are only referred for determining the shunt value, we have observed that semi-quantitative shunt assessment using visual analysis of brain uptake is pragmatic, achievable, and associated with a high success rate. Although the compelling study by Zhao et al. (4) focused only on quantitative analyses, they can test this issue as well.

We agree with Zhao et al. (4) on the important potential role of quantitative parameters derived from Tc-99m-MAA whole-body imaging in computing R-L shunting; however, some aspects need more explanation. This computation of R-L shunting is not free from limitations and might overestimate the true number of patients with shunts primarily because of the interference of unbound nuclides of free pertechnetate with uptake in the thyroid, salivary glands, and gastric mucosa during whole-body imaging. Such radiopharmaceutical impurities associated with Tc-99m-MAA may cause fluctuations in the R-L shunt percentage. Secondly, there is a logistical limitation because this technique requires longer acquisition times than brain calculation, especially in busy nuclear medicine departments.

Besides, since the majority of the included patients only had mild or moderate hepatopulmonary syndrome (HPS), the findings cannot be generalized in case of patients with severe or very severe HPS. It may bring up a question of making a comparison between 2 scintigraphic methods in terms of underlying diseases and disease severity in different cirrhotic subgroups.

Another point is considering a homogeneous group of participants to ensure an accurate comparison between studies. In this regard, attention should be paid to porto-pulmonary hypertension (PoPH) in addition to HPS, which is not uncommon in patients with chronic liver disease and/or portal hypertension. A major pathogenetic mechanism in HPS is the dilatation of the pulmonary vasculature, which leads to progressive hypoxemia due to intrapulmonary shunting. On the contrary, PoPH may be described as the obstruction of the arterial flow in the pulmonary vascular system in the presence of increased pulmonary vascular resistance, which results from high pulmonary vasoconstriction (5). Presumably, much smaller particles are required to detect R-L shunting in patients with PoPH compared to those with HPS (5).

To our knowledge, no clinical studies have performed long-term follow-up in patients with HPS diagnosed by different protocols to address their clinical outcomes. We believe that the question would be better answered by future clinical research aiming at evaluating outcomes according to shunt severity, based on methods ideally offering a more comprehensive profiling to individualize patient management.

Moreover, we would welcome future research specifically aimed at establishing or validating imaging methods for assessing HPS in patients without cirrhosis who have a better prognosis (6). Another research area is to establish or validate imaging methods to address the treatment efficacy or accurately predict outcomes. Such research would indeed be useful to clinicians when they are considering shunt assessment assuming that brain uptake can facilitate the assessment of surgical outcomes in patients with R-L shunting.
